# Decreased paraoxonase-1 activity is associated with alterations of high-density lipoprotein particles in chronic liver impairment

**DOI:** 10.1186/1476-511X-9-46

**Published:** 2010-05-14

**Authors:** Judit Marsillach, Gerard Aragonès, Bharti Mackness, Michael Mackness, Anna Rull, Raúl Beltrán-Debón, Juan Pedro-Botet, Carlos Alonso-Villaverde, Jorge Joven, Jordi Camps

**Affiliations:** 1 Centre de Recerca Biomèdica, Institut d'Investigació Sanitària Pere Virgili, Universitat Rovira i Virgili, C. Sant Joan s/n, 43201 Reus, Catalunya, Spain; 2 Department of Internal Medicine, Hospital del Mar, Institut Municipal d'Assistència Sanitària, Pg. Marítim 25-27, 08003 Barcelona, Catalunya, Spain

## Abstract

**Background:**

Paraoxonase-1 (PON1), a lactonase synthesized by the liver, circulates in blood bound to high-density lipoproteins (HDL). This enzyme is thought to degrade oxidized phospholipids and play an important role in the organism's antioxidant and anti-inflammatory system. Chronic liver diseases are characterized by decreased serum PON1 activity. The aim of the present study was to investigate the compositional changes in HDL that could influence PON1 activity in liver impairment.

**Methods:**

The study was performed in samples from five patients with advanced liver cirrhosis and with preserved renal function, chosen on the basis of having low serum PON1 activity and high serum PON1 concentration. As a control group, we accessed five healthy volunteers from among our hospital staff. Lipid and protein compositional analysis of lipoprotein particles were done by high-performance liquid chromatography, gel electrophoresis, and Western-Blot.

**Results:**

HDL particles from cirrhotic patients had an increased phospholipid content that was inversely correlated to PON1 activity. The HDL particles contained high levels of PON1 that corresponded, in part, to an immunoreactive protein of high molecular weight (55 kDa) not present in control subjects. This protein was identified as glycosylated PON1 and was also present in biopsies from patients with steatosis and from rats with CCl_4_-induced hepatic impairment. These changes were associated with an increased plasma concentration of markers of oxidative stress, inflammation and fibrogenesis.

**Conclusion:**

Abnormalities in the composition of lipids and proteins of HDL particles, including PON1 glycosylation, are associated with the decrease in serum PON1 activity in patients with liver disease. These alterations may adversely affect the protective role of HDL against oxidative stress and inflammation in these patients.

## Background

Paraoxonase-1 (PON1) is an esterase and lactonase that is found in the circulation bound to high-density lipoproteins (HDL) [1]. The original function of PON1 was that of a lactonase; lipophylic lactones constituting its primary substrates [2]. PON1 is thought to degrade oxidized phospholipids in lipoproteins and play an important role in the organism's antioxidant system [3,4]. Alterations in circulating PON1 levels have been found associated with a variety of diseases involving oxidative stress [5,6].

Experimental data show a clear link between PON1, HDL and inflammation. HDL particles possess anti-inflammatory properties, including the suppression of cytokine-induced endothelial cell adhesion molecules [7,8]. Recent studies in healthy volunteers showed a close relationship between circulating HDL levels and the inflammatory response to endotoxin challenge; the incidence and severity of clinical symptoms and the plasma concentrations of tumor necrosis factor, interleukins 1, 6 and 8, and monocyte chemoattractant protein-1 (MCP-1) being higher in subjects with low HDL than in those with normal HDL levels [9]. MCP-1 is intimately involved in the inflammatory reaction. This chemokine regulates the migration of monocytes into tissues and their subsequent differentiation into macrophages [10]. An end-product of lipid peroxidation (4-hydroxy-2-nonenal) and oxidized phospholipids present in LDL have been shown to stimulate the production of MCP-1 *in vitro *[11-13]. We had demonstrated that PON1 inhibits MCP-1 production in endothelial cells incubated with oxidized LDL, and that this property appeared to be due to its capacity to inhibit LDL oxidation [14]. Studies in experimental animals support the concept of an anti-inflammatory role for PON1. Transgenic mice fed a high-fat and high-cholesterol diet developed less atherosclerotic lesions, lower oxidative stress, and lower MCP-1 expression in their aortas than their corresponding control littermates [15]. These results confirm other studies that had shown that PON1 knock-out mice had higher peripheral lipid peroxidation and a higher degree of macrophage oxidative stress [16]. Further, a recent study showed that, even in the absence of hyperlipidemia, PON1 deficiency promoted pro-inflammatory changes in the expression of adhesins [17].

The liver plays a key role in the synthesis of PON1 [18], and chronic liver diseases are associated with increased oxidative stress, MCP-1 synthesis, and inflammation [19,20]. In previous studies, we reported that serum PON1 activity is decreased in patients with chronic liver impairment, while serum PON1 concentration and hepatic PON1 protein expression are increased [21-23]. More recent evidence indicates that PON1 over-expression provides strong protection against the development of experimental liver disease [24]. Conversely, low PON1 activity is associated with an enhanced sensitivity to the development of liver damage [25].

Not much is known about the biochemical mechanisms underlying these alterations in PON1 synthesis and secretion in liver diseases. A likely hypothesis is that PON1 activity is influenced by alterations in HDL structure and composition, secondary to an impaired synthesis of this lipoprotein by the liver. This subject has not been sufficiently explored, to-date. We performed the present study to investigate the lipid and protein changes in HDL particles that could potentially influence PON1 activity and, as well, the effect of these changes on oxidative stress and inflammation in patients with chronic liver impairment.

## Methods

### Participants

As stated before, case-control studies showing a decreased serum PON1 activity and increased PON1 concentration have already been published [21-23], and the aim of the present investigation was to add a further insight into the hypothesis that these changes may be related to other HDL compositional alterations. To maximize the observed changes, we selected five patients' samples from our bio-bank on the basis of having the lowest serum PON1 activity and the highest serum PON1 concentration. The patients (age: 46 to 76 years; 3 men, 2 women) had an advanced liver cirrhosis and preserved renal function. Their diagnoses were performed by means of a complete analytical and clinical exploration including echography to evaluate splenomegaly or portal vein dilation, and fibrogastroscopy to detect the presence of gastroesophageal varices. They all had ascites. The etiology of their disease was alcohol abuse. Serological analyses for hepatitis B, C, and human immunodeficiency virus infections were negative. As a control group, we accessed five healthy volunteers from among our hospital staff (age: 25 to 51; 3 men, 2 women). Blood samples were collected at 4°C, immediately centrifuged, and the serum or plasma frozen at -80°C for batched analyses. For some of the Western blot analyses described in the Results section we obtained liver biopsy material from 2 patients with hepatic steatosis and inflammation, who had undergone bariatric surgery for the treatment of morbid obesity. The study was approved by the Ethics Committee of the Hospital Universitari de Sant Joan and written informed consent was obtained from all the participants.

### Induction of experimental liver damage in rats

To investigate the differential expression of PON1 protein in normal and diseased livers, we employed the model of CCl_4_-induced hepatic damage in rats [26]. Liver fibrosis was induced in male Wistar rats weighing 207 ± 9 g (Panlab, Barcelona, Spain) by twice-a-week intra-peritoneal (i.p.) injections over a period of 6, 8 and 12 weeks (2 animals for each time period) of 0.5 mL of CCl_4 _diluted 1:1 (v/v) in olive oil. An additional group of 2 rats receiving only the excipient olive oil was used as a control group. Fibrosis and cirrhosis were efficiently induced in CCl_4_-administered rats, as verified by histopathology. All the animals were fed *ad libitum *with standard rat chow (Harlan Interfauna, Barcelona, Spain). Before sacrifice, the livers were removed under anesthesia, portions were frozen in liquid nitrogen, and stored at -80°C for subsequent analyses. The handling of animals and the procedures described were approved by the Ethics Committee on Animal Experimentation of the Rovira i Virgili University.

### Compositional analysis of serum lipoproteins

Human serum lipoproteins were separated by a dual detection high-performance liquid chromatography method (HPLC) as previously described [27,28]. In brief, 100 μl of diluted serum were applied onto two columns of TSK gel Lipopropak XL connected in tandem and eluted at a flow rate of 0.7 ml/min with 50 mM Tris acetate buffer, pH = 8.0, containing 0.3 M sodium acetate, 0.05% sodium azide, and 0.005% Brij-35. The TSK column medium is composed of porous polymer matrices with a nominal bed size of 10 μm and a pore size of 100 nm. The matrices are expected to exclude most of chylomicrons in the void volume. The concentrations of total cholesterol, free cholesterol, triglycerides, and phospholipids in serum and in the eluted fractions were measured by enzymatic methods (Wako Chemicals, Osaka, Japan). The concentrations of apolipoproteins (apo) A-I, apo A-II and apo E were determined by immunoturbidimetry (Daiichi Pure Chemicals Co., Tokyo, Japan). Lipoprotein fractionation by HPLC and the measurement of lipid and apolipoprotein concentrations were outsourced to Skilight Biotech Inc. (Tokyo, Japan). The lipoprotein fractions were then returned frozen via door-to-door express courier to Reus for the measurement of PON1 activity and concentration. PON1 activities in serum and lipoprotein fractions were measured as the rate of hydrolysis of paraoxon at 410 nm and 37°C [21]. PON1 concentration was determined by an in-house enzyme-linked immunosorbent assay (ELISA) method [29].

### Western blot of HDL-bound and liver PON1

HDL isolation was performed by the method of Havel et al. [30] in a Centricon 75 ultracentrifuge (Kontron Instruments Ltd., Italy) using a Kontron TFT 45.6 fixed angle rotor. We performed a denaturing gel electrophoresis with HDL protein load of 20 μg, or 25 μg of liver protein, in a NuPAGE^® ^4-12% pre-cast gel (Invitrogen, Carlsbad, CA, USA). Because lipids modify the electrophoretic mobility of the proteins, HDL were previously delipidated with methanol and diethyl ether, and resuspended in urea. Liver homogenates were obtained as previously described [31]. Protein transfer on to a nitrocellulose membrane was with an iBlot™ Dry Blotting System (Invitrogen). PON1 Western blot was performed with antibodies obtained by inoculating rabbits with the peptide CRNHQSSYQTRLNALREVQ derived from the sequence of mature PON1, as has been described [29,32]. The antibody was used at a 1/10000 dilution. A polyclonal anti-rabbit-HRP secondary antibody (Dako, Glostrup, Denmark) was used at a dilution of 1/2000. We used the Amersham ECL™ Advanced Western Blotting Detection Kit (GE Healthcare, Fairfield, CT, USA) for the chemiluminescent detection of the bands. The images were acquired with a Versadoc Gel Imaging System (Bio-Rad, Hercules, CA, USA).

To characterize the glycosylation status of PON1 immunoreactive bands, we incubated HDL with peptide-N-glycosidase F (PNGase F), according to the manufacturer's instructions (Sigma, St. Louis, MO). This enzyme releases asparagine-linked oligosaccharides from glycoproteins by hydrolyzing the amide group of the asparagine side chain.

### Gel electrophoresis of HDL particles

HDL particle size was estimated by non-denaturing gel electrophoresis of the HDL fraction obtained by ultracentrifugation. A polyacrylamide gel (Novex^® ^4-20% Tris-Glycine pre-cast gels; Invitrogen) was run according to the manufacturer's indications. The bands were visualized by Coomassie^® ^blue staining (Colloidal Blue Staining Kit; Invitrogen).

### Measurement of other biochemical parameters

Serum cholesteryl ester transfer protein (CETP) was analyzed by the method of Lagrost and Barter [33]. Lecithin:cholesterol acyl transferase (LCAT) activity was measured by the method of Lagrost et al. [34]. Phospholipid transfer protein (PLTP) was determined by the method of Damen et al. [35]. Serum total peroxides concentration was analyzed by a colorimetric enzymatic assay (Immun-Diagnostik AG, Benshein, Germany). MCP-1 concentration in EDTA plasma was measured by ELISA (Peprotech, London, UK). Serum type III procollagen-N-peptide (P-III-P) concentration, a marker of liver fibrogenesis, was determined by radioimmunoassay (Orion Diagnostica OY, Espoo, Finland). Serum alanine aminotransferase, aspartate aminotransferase, γ-glutamyl transferase, and alkaline phosphatase activities, and albumin and bilirubin concentrations were measured by standard methods with reagents purchased from Beckman-Coulter (Fullerton, CA, USA).

### Statistical analysis

Curvilinear regression analyses were employed to evaluate the degree of association between variables. Results are shown as means and SD (in parenthesis). Statistical analyses were performed with the SPSS 17.0 statistical package (SPSS Inc., Chicago, IL. USA).

## Results

### Changes in liver function tests and serum lipoproteins

**Figure 1:**
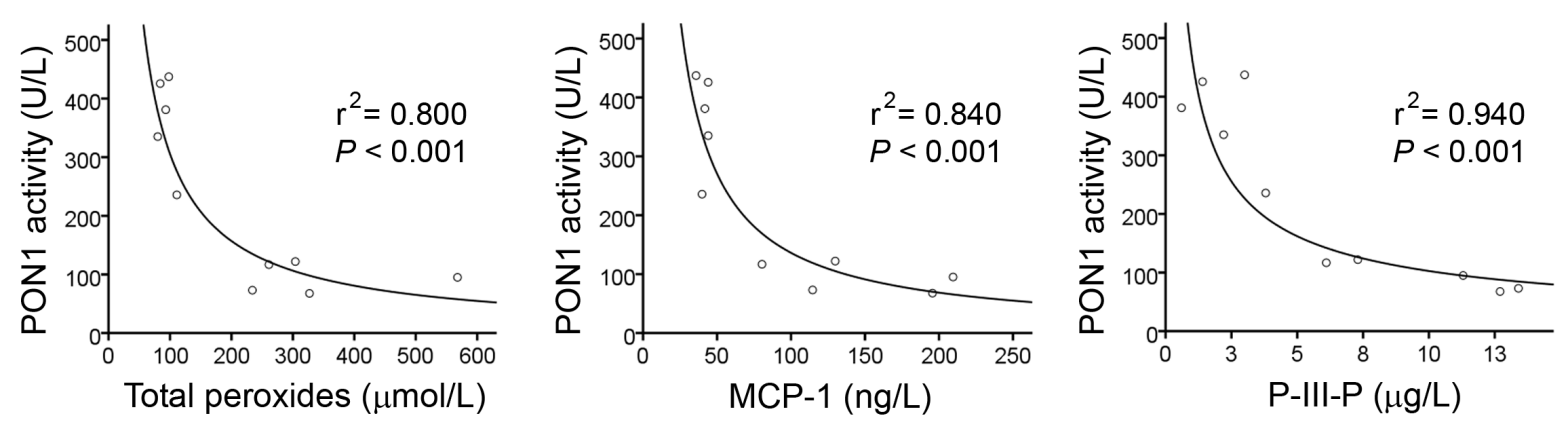
**Relationships between serum PON1 activity, total peroxides, and MCP-1 and P-III-P concentrations**.

Relative to the control group, the patients with chronic liver impairment had, as expected, a significant increase in serum aminotransferases and alkaline phosphatase activities together with a decrease in albumin, HDL-cholesterol, and apo A-I concentrations. They also showed a profound decrease in serum PON1 activity and an increase in PON1 concentration, together with an increase in total peroxides, MCP-1, and P-III-P concentrations (Table 1). There were highly significant inverse relationships between PON1 activity and total peroxides, MCP-1, and P-III-P (Fig. 1).

**Table 1:** Serum liver function tests, lipid and apolipoprotein levels in control subjects and patients with liver cirrhosis.

Variable	Control subjects	Patients	*p*
Albumin; g/L	41.5 (1.6)	36.6 (9.1)	0.005
Alanine aminotransferase; μkat/L	0.31 (0.08)	1.08 (0.70)	0.041
Aspartate aminotransferase; μkat/L	0.35 (0.06)	1.57 (0.99)	0.025
γ-glutamyl transferase; μkat/L	0.39 (0.27)	1.19 (1.06)	0.141
Alkaline phosphatase; μkat/L	0.79 (0.25)	5.81 (4.11)	0.026
Bilirubin; μmol/L	12.8 (1.9)	19.4 (16.1)	0.410
Total peroxides: μmol/L	93.2 (12.2)	338.8 (133.2)	0.003
Monocyte chemoattractant protein-1; ng/L	41.1 (3.4)	146.0 (54.9)	0.003
Type III procollagen-N-peptide; μg/L	2.2 (1.3)	10.2 (3.3)	0.001
Paraoxonase-1 activity; U/L	362.9 (36.6)	94.9 (11.0)	< 0.001
Paraoxonase-1 concentration; mg/L	62.1 (25.5)	189.3 (48.3)	0.002
Cholesterol; mmol/L	5.26 (1.29)	4.24 (2.00)	0.366
Free cholesterol; mmol/L	1.37 (0.36)	1.48 (0.76)	0.795
Triglycerides; mmol/L	1.26 (0.19)	1.31 (0.54)	0.843
Phospholipids; mmol/L	1.38 (0.36)	1.48 (0.76)	0.795
HDL-cholesterol; mmol/L	1.80 (0.50)	1.19 (0.27)	0.044
Apolipoprotein A-I; g/L	1.41 (0.40)	0.89 (0.27)	0.050
Apolipoprotein A-II; g/L	0.31 (0.11)	0.22 (0.11)	0.234
Apolipoprotein E; mg/L	25.59 (5.37)	39.20 (24.62)	0.262

Values are expressed as means and SD (in parenthesis).

**Figure 2:**
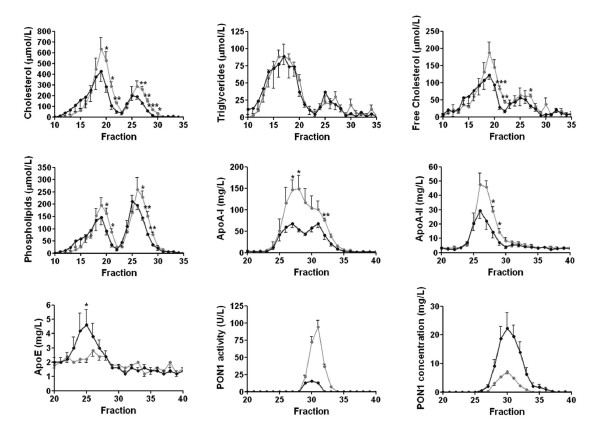
**Compositional analysis of serum lipoproteins in the control subjects (gray lines) and cirrhotic patients (black lines)**.

We observed significant changes in the HPLC lipoprotein profile in the patients with respect to the control group (Fig. 2). Patients had significant decreases in cholesterol and phospholipid concentrations in very low-density and low-density lipoproteins (VLDL + LDL, fractions 10 to 22) and in HDL (fractions 23 to 30). Free cholesterol was also decreased in triglyceride-rich lipoproteins, but not in HDL. We did not observe any significant changes in triglyceride concentrations. Apo A-I concentrations were markedly decreased in the patients. The results indicated two peaks which reflect two different HDL subpopulations with compositional differences. The peak between fractions 25 and 28 contained apo A-II (which was significantly decreased in the patients) and apo E (which was significantly increased), but not PON1. The peak between fractions 29 and 35 did not contain any apo A-II nor apo E, but contained PON1 enzyme activity and protein mass. PON1 activity was significantly decreased, and PON1 concentration was significantly increased in the patient group relative to controls.

**Figure 3:**
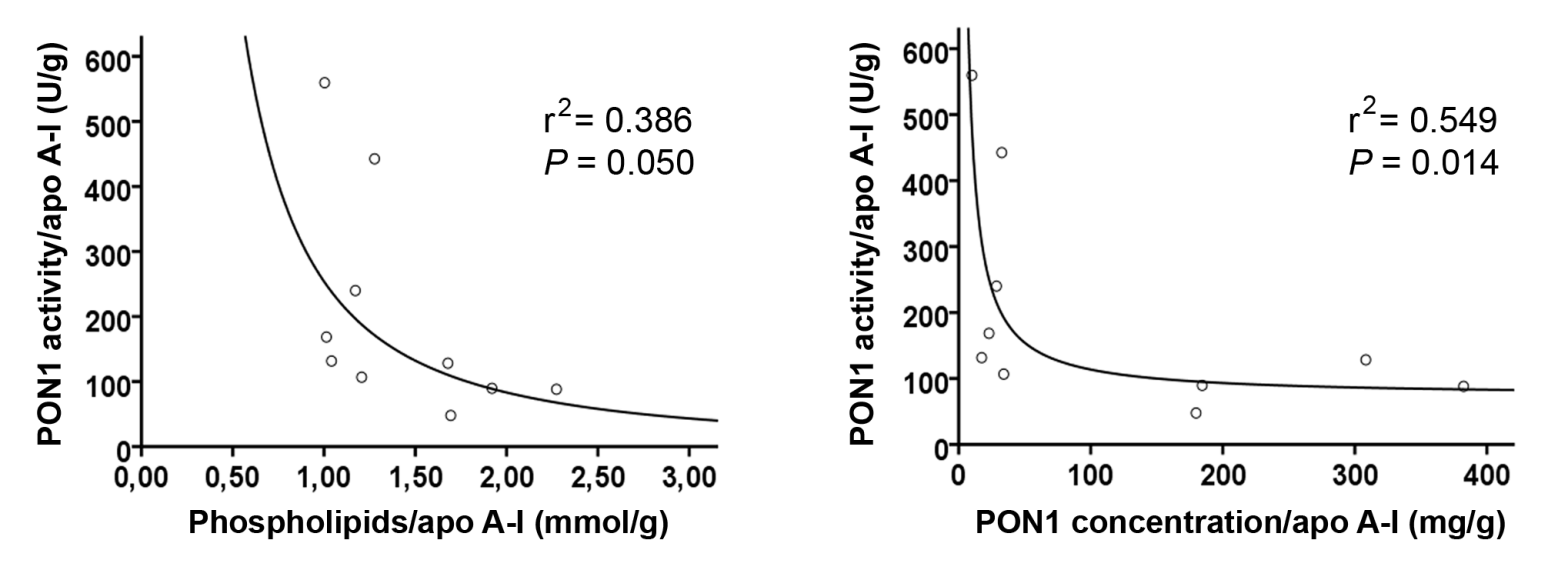
**Relationships between PON1 activity and concentration, and phospholipids in HDL particles**. In calculating the ratios, the values of the measured variables in HPLC fractions 29 to 35 were summed and divided by the sum of apo A-I values in the same fractions.

Because changes in HPLC profile do not indicate whether the observed changes are due to compositional changes in HDL particles or to a reduced number of particles, we calculated the measured variables as ratios of apo A-I concentrations. We observed that patients with liver impairment had a significant increase in the HDL phospholipids to apo A-I ratio [1.75 (0.39) vs. 1.10 (0.12); *p *= 0.007] and in the PON1 mass to apo A-I ratio [227.5 (93.1) vs. 45.0 (10.1); *p *= 0.002]. There were no significant differences in any of the other ratios. The phospholipids to apo A-I ratio and the PON1 protein to apo A-I ratio were inversely correlated with PON1 activity (Fig. 3).

### Abnormal immunoreactive protein reaction against anti-PON1 antibodies

**Figure 4:**
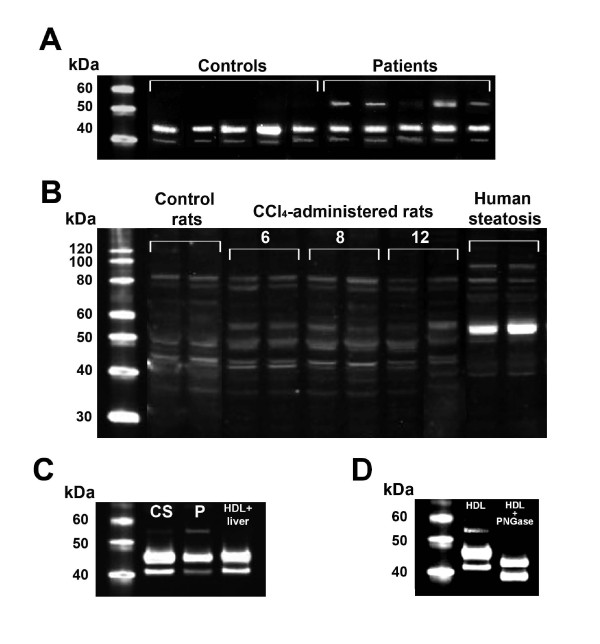
**(A) Western blot analysis of HDL particles in control subjects and patients with liver cirrhosis**. HDL from the patients had an immunoreactive band (very light in the case #3) of about 55 kDa. (B) Western blot analysis of liver homogenates from control rats, CCl_4_-administered rats over a period of 6, 8 and 12 weeks, and liver biopsy samples from human patients with liver steatosis. A band at the relative molecular weight of 55 kDa increased progressively over the time-course of CCl_4 _administration. A similar, very intense band was observed in patients with steatosis. (C) Western blot analysis of HDL particles from a control subject (CS), a patient with cirrhosis (P), and normal HDL incubated *in vitro *with a liver homogenate from a patient with steatosis. (D) Western blot analysis of HDL particles from a patient with liver cirrhosis, with and without pre-incubated with PNGase F.

The HDL particles from cirrhotic patients contained high levels of a protein which reacted against anti-PON1 antibody and had an estimated weight of 55 kDa. This protein was not present in the control subjects (Fig. 4A). To further characterize this protein, we performed Western blot analysis of the liver homogenates of biopsies obtained from patients with hepatic steatosis, as well as from rats with CCl_4_-induced hepatic impairment. We observed that the livers from both sources, the patients and the CCl_4_-administered animals, had a similar immunoreactive band (Fig. 4B). However, when a liver homogenate from a patient with steatosis was incubated *in vitro *with normal human HDL and then subjected to Western blot analysis, we did not observe any abnormal immunoreactive band (Fig. 4C). Further, this protein band disappeared when HDL was incubated with PNGase F; suggesting that it consisted of glycosylated PON1 (Fig. 4D).

### Changes in HDL particle size and in enzymes related to HDL synthesis

**Figure 5:**
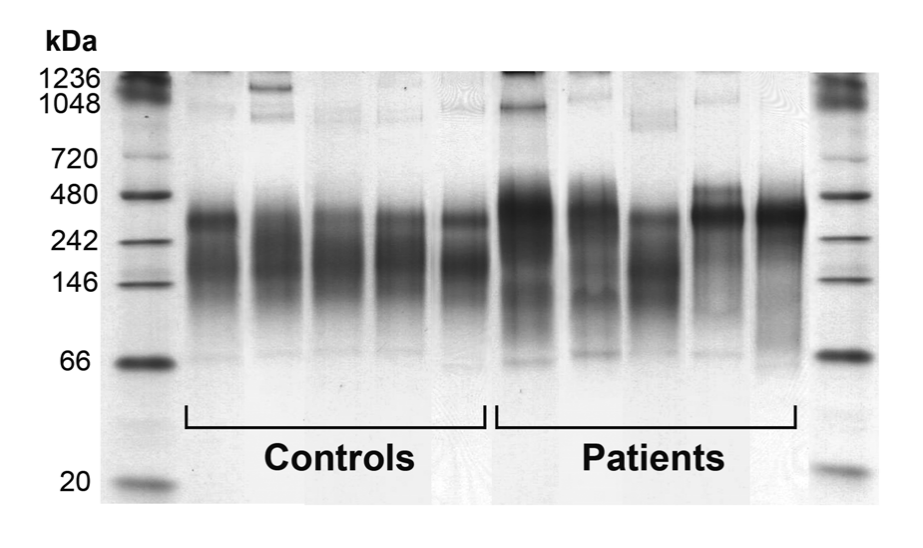
**Non-denaturing electrophoresis of HDL particles**.

We investigated the alterations in HDL particle size using non-denaturing gel electrophoresis. Patients with chronic liver disease had a higher degree of heterogeneity with respect to HDL particle size than healthy volunteers (Fig. 5). Electrophoretic banding patterns indicated the presence of small (<100 kDa) HDL particles only in the patients. HDL particles between 100 and 200 kDa were present in the sera of all five control individuals but in only two of the patients. On the other hand large (>300 kDa) HDL were present in all five patients, but in only two of the control subjects. We observed a significant increase in serum CETP and PLTP, and a decrease in LCAT activities in patients relative to controls (Table 2).

**Table 2:** Serum CETP, PLTP and LCAT activities in control subjects and patients with liver cirrhosis.

Variable	Control subjects	Patients	*p*
CETP; μmol/hr^-^·L	180.9 (27.3)	292.3 (67.1)	0.009
PLTP; mmol/hr·L	1.71 (0.27)	3.48 (1.28)	0.017
LCAT; μmol/hr·L	17.9 (2.6)	11.4 (5.3)	0.049

Values are expressed as means and SD (in parenthesis).

## Discussion

The molecular bases underlying the alterations in the circulating PON1 activity together with its synthesis and secretion in chronic liver diseases are far from being completely understood, despite these changes being of crucial importance in the pathophysiology of chronic liver impairment. As illustrated in the present investigation, patients with liver cirrhosis had an increased serum concentration of total peroxides (a marker of oxidative stress), MCP-1 (an index of inflammation), and P-III-P (a marker of liver fibrogenesis). All these changes were observed to be strongly associated with a decrease in serum PON1 activity. Although a direct functional relationship between serum PON1 activity alterations, inflammation and fibrogenesis cannot be deduced from the present investigations, these data provide further support for studies showing that oxidative stress activates hepatic stellate cell and macrophages *in vitro *and in experimental models of liver impairment [32,36-38]. A possible answer to the question of why serum PON1 activity is decreased is that PON1 is inactivated by oxidized lipids, as was shown by Aviram et al. [39] who demonstrated that the incubation of PON1 *in vitro *with oxidized palmitoyl arachidonoyl phosphatidylcholine, lysophosphatidylcholine, oxidized cholesteryl arachidonate and oxidized LDL resulted in inactivation of PON1 arylesterase activity. Indeed, in a previous study, we observed that alcoholic patients with a normal, or with minimally-affected liver function, already had a decreased serum PON1 activity that was associated with an increase in the circulating concentration of malondialdehyde [23]. These data support the hypothesis of a direct inhibition by lipid peroxides on the PON1 enzyme active site. However, liver cirrhosis is a chronic and diffuse disease that is usually protracted in its development, and in which multiple metabolic derangements appear progressively, including an altered capacity of pan-protein synthesis by the liver. A likely possibility (and one which does not exclude an inhibition by lipid peroxidation products) is that, in advanced liver impairment, changes in HDL structure and composition influence PON1 activity. This appears feasible since it is well documented that PON1 activity is profoundly dependent on the lipid and protein compositional environment of the HDL particles [40].

Results from the present study suggest that PON1 activity and mass are associated, both in control subjects as well as in cirrhotic patients, with a specific sub-population of HDL particles containing apo A-I, but with not apo A-II and apo E. These results agree with a study by Cabana et al. [41] that showed a similar distribution of PON1 in human HDL sub-fractions obtained by FPLC and with experimental studies in apo A-II transgenic mice in which apo A-II enrichment was found to displace PON1 from HDL and, as well, to impair the antioxidant and athero-protective function of this particle [42,43]. However, our results differ from other reports in which HDL was isolated by other methods. Bergmeier et al. [44] observed a high PON1 activity in HDL_3 _particles isolated by ultracentrifugation, and which contained apo E. Moren et al. [45] observed considerable PON1 activity in apo A-II-containing HDL isolated by immunoprecipitation. Methodological differences probably account for the observed discrepancies, but we believe that HPLC or FPLC fractionation methods are less physico-chemically aggressive and, as such, are less likely to alter HDL composition in the process of isolation.

Non-denaturing gel electrophoresis showed a considerable increase in very small and very large HDL particles from cirrhotic patients compared to the control subjects. These changes are probably secondary to alterations in CETP, PLTP, and LCAT levels in these patients. Although reduced LCAT activity is common in a variety of liver diseases [46], the current report is, to the best of our knowledge, the first to identify increased CETP and PLTP activities as being associated with chronic liver impairment. These findings will require confirmation in much larger studies of cases *versus *controls. The mechanisms leading to increased CETP and PLTP activities in chronic liver disease are also unclear at present. The outcome observed could be due to increased synthesis and secretion, decreased degradation (or both) or an entirely different mechanism such as an effect of HDL composition on enzyme activity. Again, further studies are required. The combination of decreased LCAT and increased CETP activities in the patients probably resulted in the increase in small HDL particles. However, the greater PLTP activity would also have resulted in increased fusing of small HDL into the larger HDL particles found in the patients [47-49]. Therefore changes in the activities of the lipid transfer proteins could explain the different pattern of HDL particles found in the patients. An increase in PLTP would lead to larger HDL particles, while an enhancement in CETP and a decrease in LCAT would result in smaller HDL particles with an increase in the HDL_3 _sub-fraction. While the impact of a larger particle size in relation to HDL function remains unclear, extensive data from the literature show that small HDL particles lose most of their antioxidant properties, and may become pro-oxidant and pro-inflammatory instead [50,51]. In addition, results from the present study suggest that these small particles do not carry PON1 and, as such, would contribute to the decrease in serum PON1 activity observed. However, it is also possible that changes in HDL size are associated with firmer binding of PON1 but not with changes in activity.

We noted an inverse relationship between phospholipid content and PON1 activity in HDL particles. In addition, considerable amounts of PON1 protein, but low enzyme activity, were observed in particles eluting at HPLC fractions #31-35 in which phospholipids concentrations were below the detection limit of the assay. These results suggest that an appropriate balance between phospholipids and PON1 is important in maintaining the enzyme's activity. Indeed, the proportional distribution of phospholipids and apo A-I in HDL has been shown to play an important role in PON1 secretion, stability and activity. Sorenson et al. [52] showed that PON1 was associated with HDL more via the binding to phospholipids than to apo A-I. Nevertheless, apo A-I stabilized the enzyme activity more than did phospholipids alone. These results were confirmed by Deakin et al. [53]. Hence the hypothesis follows that an increased phospholipids to apo A-I ratio, as observed in cirrhotic patients, contributes to PON1 instability. Previous studies from our group [22,23,31] showed that PON1 activity in patients with liver disease is inversely related to PON1 mass. These results prompted us to investigate the nature of PON1 protein in patients with cirrhosis. One of the more interesting findings from the present investigation has been the identification by Western blot of an abnormal protein that reacted against the anti-PON1 antibody, had a higher molecular weight than PON1, and was present in the HDL from cirrhotic patients but not in control subjects. This protein was also present in the livers of patients with steatosis (a more benign form of liver disease) and in rats with CCl_4_-induced cirrhosis. Additionally in these animals, the amount of abnormal PON1 increased along the time-course of the disease development. These results highlight the presence of this protein in liver impairment *per se*, irrespective of the etiology or, even, the animal species. The incubation of HDL with PNGase F strongly suggests that this protein is, indeed, a highly glycosylated form of PON1. Interestingly, Liu et al. [54] observed that when they produced recombinant human PON1 in a baculovirus system in Hi5 insect cells, a significant proportion of PON1 was synthesized as a protein of approximately 54,000 Da, and which they identified as a PON1 multimer. Taking these data together, we can hypothesize that abnormal PON1 molecules are synthesized under diverse altered conditions such as liver disease, or when human PON1 is synthesized by phylogenetically-distant cells. An important finding of the present study is that when a liver homogenate from a patient with steatosis was incubated *in vitro *with normal HDL, the altered immunoreactive band was no longer observed. These results suggest that the high molecular weight PON1-like protein is actively incorporated into the HDL particles within the hepatocytes, and that it is not a mere artifact or a result of a non-specific adsorption of PON1 fragments by HDL particles in the circulation. The possibility exists that highly glycosylated PON1 is abnormally assembled into HDL particles in liver disease secondarily to alterations in protein synthesis in hepatocytes or to altered hepatic lipid metabolism.

## Conclusions

We conclude that abnormalities in the composition of lipids and proteins of HDL particles are associated with the decrease in serum PON1 activity in patients with liver disease. Of special note is the presence of a high molecular weight inactive PON1 carried in the HDL in large quantities. These alterations may play a role in the diminished protection against oxidative stress and inflammation in these patients.

## Abbreviations

Apo: apolipoprotein; CETP: cholesteryl ester transfer protein; ELISA: enzyme-linked immunosorbent assay; FPLC: fast-protein liquid chromatography; HDL: high-density lipoproteins; HPLC: high-performance liquid chromatography; LCAT: lecithin:cholesterol acyl transferase; LDL: low-density lipoproteins; MCP-1: monocyte chemoattractant protein-1; PLTP: phospholipids transfer protein; PNGase: peptide-N-glycosidase; PON1: paraoxonase-1; P-III-P: type III procollagen-N-peptide; VLDL: very low-density lipoproteins.

## Competing interests

The authors declare that they have no competing interests.

## Authors' contributions

JM, JP-B, CA-V, JJ and JC had substantial contributions to conception and design, statistical analysis and writing the manuscript. JP-B and CA-V recruited the subjects of the study. JM, BM, MM, AR, RB-D performed the analytical measurements. All authors read and approved the final manuscript.
